# Idiopathic Isolated Acquired Steroid Dependent SO Palsy: A Rare Case Report

**DOI:** 10.1155/2019/4812380

**Published:** 2019-01-23

**Authors:** Isha Agarwal, Mayuresh Naik, HarinderSingh Sethi

**Affiliations:** Department of Ophthalmology, V.M.M.C. & Safdarjung Hospital, Ring Road, Ansari Nagar, New Delhi 110029, India

## Abstract

15-year-old boy presented with sudden onset, stable, nonprogressive painless diplopia (greatest in right gaze and inferior field of view) and hyperdeviation of left eye for a year. On ophthalmic examination, the patient had uncrossed diplopia with tilt and separation maximum in dextrodepression. On Park's three step test, left eye hypertropia increased on right gaze and left tilt suggestive of left superior oblique (SO) palsy. On prism bar cover test (PBCT), deviation was more than 25 PD base-down over the left eye for both distance and near in all gazes. MRI head and orbit revealed a normal study while the myasthenia and inflammatory work-up was unremarkable. A provisional diagnosis of “Idiopathic Acquired Left Superior Oblique Palsy” was made and the patient was given trial of oral steroids at 1 mg/kg body weight. At 6 weeks, patient's diplopia resolved and PBCT neutralised at 6PD. Oral steroids were gradually tapered off by 10 mg per week with weekly follow-up. Upon decreasing the dose of prednisolone to 5 mg, intermittent diplopia and 18 PD left hypertropia reappeared. When patient was again restarted on oral steroids at 1 mg/kg body weight, diplopia-hypertropia disappeared at 10 mg OD prednisolone only to reappear at 5 mg OD dosage, leading to the final diagnosis of a “Steroid Dependent Isolated Superior Oblique Palsy”. Presently, the patient is maintained on a daily dose of 10 mg oral prednisolone.

## 1. Case Report

A 15-year-old boy presented with sudden onset painless diplopia and hyperdeviation of left eye for almost a year, which was stable, painless, and nonprogressive. The binocular diplopia persisted both for near and for distance and was greatest in right gaze and inferior field of view.

There was no history of any associated vision loss, pain, trauma, febrile illness, or any other systemic illness. There was neither any history of weakness after prolonged work or in the evenings nor any past history of strabismus or squinting for far.

There was no history of any weakness, of decreased sensations in body part, of tremors or jerks, of sudden severe pain, of neck stiffness, or of loss of consciousness at the onset of the symptoms. There were no associated neurological symptoms including no other cranial nerve abnormalities. The patient's past medical history and birth history were uneventful. Also there was no history of similar complains among family members.

General physical examination and systemic review were unremarkable.

**Figure 1:**
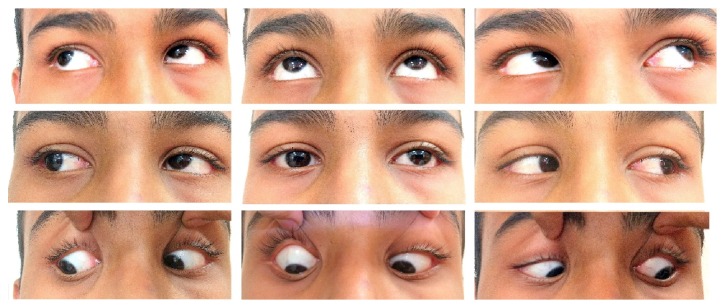
Clinical photograph of 9 cardinal gazes at presentation.

On ophthalmic examination, the Best Corrected Visual Acuity (BCVA) in both the eyes was 6/6 Snellen's for distance and J6 Jaeger's for near. The patient had a compensatory head posture with the chin at level, face turned towards the right side, and a head tilt to right. Extra ocular movements were full and free in both the eyes with inferior oblique over action in left eye (Figure 1). On diplopia charting, patient had uncrossed diplopia with tilt and separation maximum in dextrodepression. Park-Bielschowski's three step test suggested a left superior oblique (SO) palsy. On prism bar cover test (PBCT) with prism over the left eye, the deviation was more than 25 PD base-down for both distance and near in all cardinal gazes. Hess charting corroborated the clinical findings. Forced duction test (FDT) and force generation testing (exaggerated FDT) were carried out in an ICU setting and revealed neither any restriction of movement nor laxity of muscles in any gaze.

Revisiting old photographs did not reveal any plagiocephaly or facial asymmetry, thus negating a congenital deviation. Hematological investigations also did not reveal any anaemia, thyroid dysfunction, or any other abnormality.

**Figure 2:**
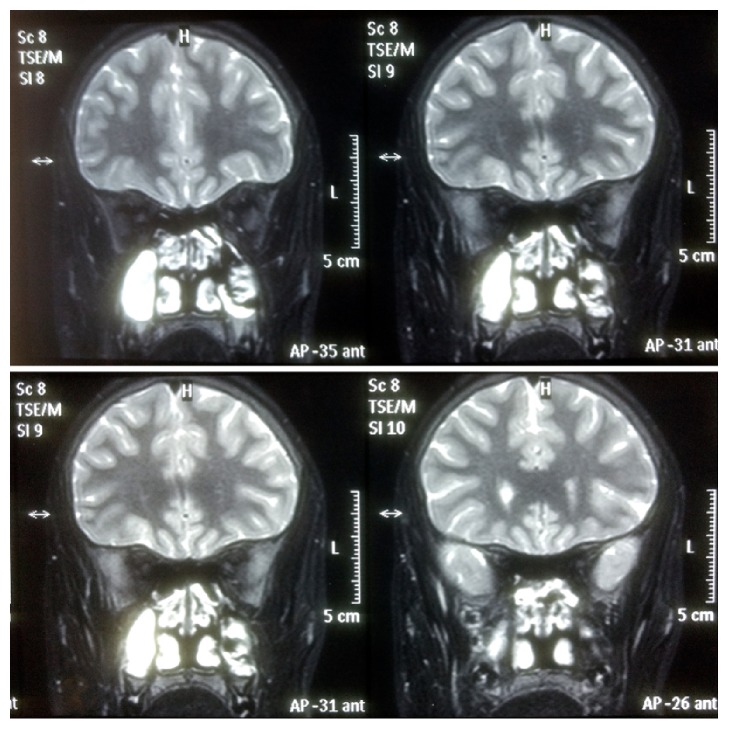
MRI head and orbit coronal section at the level of the midorbit and apex showing absence of any granulomatous lesions nor any extraocular muscle thickening (ruling out myositis or orbital pseudotumor).

**Figure 3:**
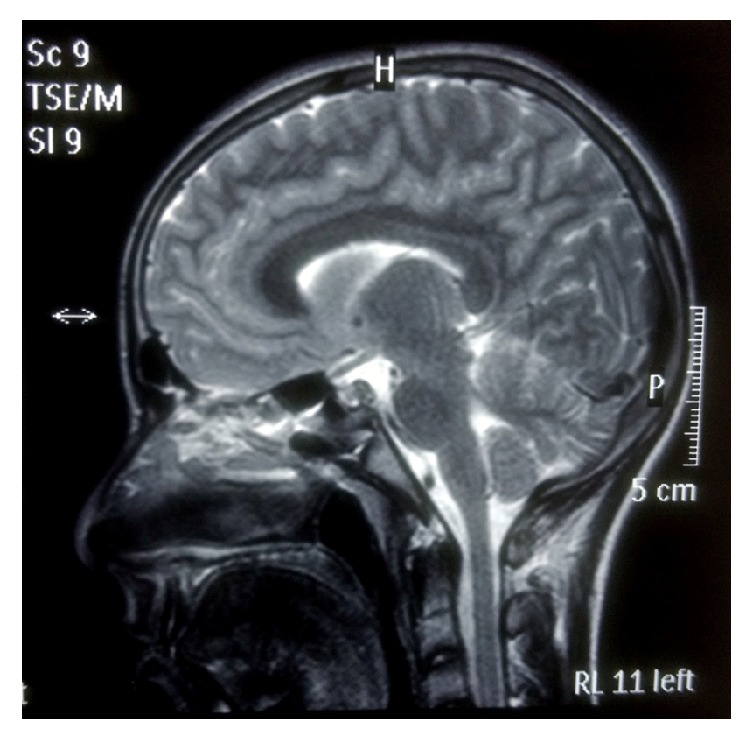
MRI head and orbit at the level of the midorbit and apex showing absence of any granulomatous lesions nor any extraocular muscle thickening (ruling out myositis or orbital pseudotumor).

Ultrasound (USG)-B scan of both the eyes was unremarkable. Gadolinium enhanced MRI of head and orbit was normal, neither any inflammatory lesions in the orbit or the cavernous sinus nor any demyelinating plaques in the periventricular areas (Figures 2 and 3). Anti-AchR and anti-MUSK antibodies were negative and single fiber electromyography (SF-EMG) was within normal limits, thus ruling out the possibility of ocular myasthenia. Blood count and thyroid functions were normal. An inflammatory work-up revealed normal ESR and CRP and negative RF, ANA, cANCA, and pCNCA. A provisional diagnosis of “Idiopathic Acquired Superior Oblique Palsy” was made.

**Figure 4:**
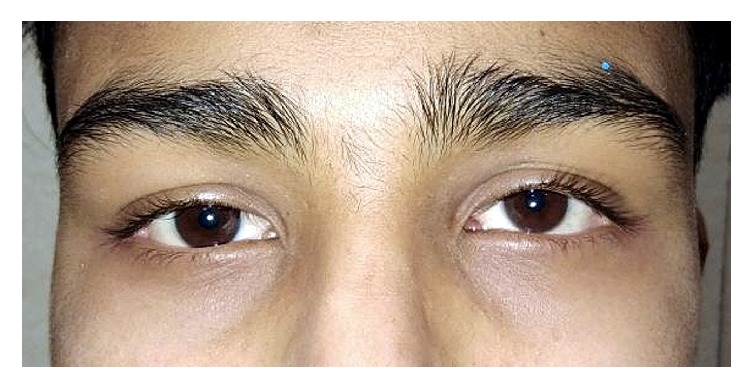
Clinical photograph of patient after 4 weeks of 1 mg/kg oral steroid.

The patient was given a trial of oral steroids, i.e., Prednisolone 1 mg/kg body weight. After 2 weeks, the patient was symptomatically better, now with only intermittent diplopia in inferior gaze and decrease in the upward deviation of left eye according to patient's father. PBCT for both near and distance (with prism over the left eye) neutralised the hypertropia with 18 PD base-down over left eye. 4 weeks after starting the oral steroids, the patient's diplopia resolved the left hyperdeviation measured 6PD. After 6 weeks of treatment, the patient remained free of diplopia-hypertropia. Oral steroids were gradually tapered off by 10 mg per week and weekly follow-up was done (Figure 4). Upon decreasing the dose of prednisolone to 5mg per day, the intermittent diplopia and 18PD left hypertropia reappeared. Oral steroids were restarted at 1 mg/kg body weight and in 2 weeks the diplopia had resolved again and the left hypertropia decreased to 6PD. When the steroid dose was again gradually tapered, intermittent diplopia and 16PD hypertropia reappeared upon reducing the dose to 5 mg per day.

The oral steroid dose was increased back to 10 mg per day and after a week the diplopia disappeared and PBCT neutralised at 6PD base-down in front of left eye.

This unique sequence of events, i.e., disappearance of diplopia-hypertropia at 10 mg OD prednisolone and reappearance at 5 mg OD dosage, led to the final diagnosis of a “Steroid Dependent Isolated Acquired Superior Oblique Palsy”. Presently the patient is maintained on a daily dose of 10 mg oral prednisolone.

## 2. Discussion

The trochlear nerve, also known as the fourth cranial nerve, arises from the trochlear nucleus, situated on the dorsal aspect of the midbrain below the inferior colliculus just lateral to the frenulum veli [1, 2]. It is the only cranial nerve that arises from the dorsal aspect of the brain resulting in the longest intracranial course of any cranial nerve and has the fewest axons of all the cranial nerves, making it slender and vulnerable to trauma. It is the only decussated cranial nerve apart from optic nerve, thus innervating the contralateral superior oblique muscle. Nuclear lesions cause contralateral SO palsy and peripheral lesions cause ipsilateral SO palsy [3].

The most common etiology of isolated 4th nerve palsy is congenital, contributing to around 39.5% of the cases, closely followed by traumatic (34%), idiopathic (23.2%), and neurologic (2.9%) paralyses [4, 5].

Congenital fourth nerve palsy often remains compensated and unrecognized during childhood due to large vertical fusional vergence amplitudes and can manifest later either spontaneously or after a minor trauma [1].

Trauma commonly leads to bilateral fourth nerve palsy due to injury to the decussating nerve fibers at the anterior medullary velum. Other etiologies of acquired causes of 4th nerve palsy include compressive lesions (aneurysms and tumors), demyelination, ocular myasthenia, diabetic neuropathy, and herpes zoster. Around 20% cases are idiopathic [6–8]. 4th nerve palsy of microvascular etiology is usually seen in older patients with vascular risk factors.

Several syndromes (superior orbital fissure syndrome, orbital apex syndrome, and cavernous sinus syndrome) cause trochlear nerve palsy associated with variable involvement of cranial nerves 2nd through 6th. However, our patient presented with isolated 4th nerve palsy.

For an isolated nonprogressive 4th nerve palsy, neuroimaging is not required routinely; however imaging is recommended if there is any neurologic deficit, in acute traumatic cases and in patients with unclear etiology [9, 10]. The recently established guideline for the cost-effective evaluation of patients with SO palsy suggests that isolated congenital or old traumatic cases may not require neuroimaging [11]. In most cases of 4th nerve palsy, detailed evaluation with an elaborate history and thorough clinical examination and high index of suspicion reveal the etiology. According to a recent study, neuroimaging is now recommended in all patients presenting with acute onset isolated ocular motor cranial nerve palsy [10, 12–15].

Pain is an important indicator of etiology. Painful SO palsy occurs in traumatic cases, neurological syndromes, extraocular muscle (EOM) myositis, nonspecific idiopathic orbital inflammatory disorders (NSIOID), Graves ophthalmopathy, and cysticercosis with microscopic leaks. Painless SO palsy more commonly occurs in congenital cases, myasthenia, chronic progressive ophthalmoplegia CPEO, demyelination, and nuclear palsies.

In our patient, there was no improvement in vertical diplopia or hypertropia over a year and further investigations did not reveal any abnormality. Since there was conspicuous absence of pain throughout the duration of the diplopia, a differential diagnosis of ocular myasthenia, demyelinating lesion (?multiple sclerosis), and central inflammatory lesion (vasculitis) was considered. Gadolinium enhanced MRI imaging of the head and orbit was done looking for any inflammatory or demyelinating lesion, both of which were absent. AntiAChR and antiMuSK antibodies were negative. Extensive inflammatory work-up was unremarkable so that other autoimmune diseases were ruled out. Thereafter a trial of oral steroids [10] was administered, to which the patient responded very well. However to our surprise, upon withdrawal of steroids after a tapering regime, the diplopia and hypertropia recurred. Taking into account the systemic side effects of long term oral steroids, steroid sparing immunosuppressive agents are considered for the patient.

To the best of our knowledge after a thorough review of literature, such a clinical presentation has not been reported previously and this is the first instance of a “Steroid Dependent Isolated Acquired Superior Oblique Palsy”.
